# Decreased lncRNA, TINCR, promotes growth of colorectal carcinoma through upregulating microRNA-31

**DOI:** 10.18632/aging.103436

**Published:** 2020-07-17

**Authors:** Zhong Ren, Jingzheng Liu, Jian Li, Liqing Yao

**Affiliations:** 1 Endoscopy Center, Endoscopy Research Institute, Zhongshan Hospital, Fudan University, Shanghai 200032, China

**Keywords:** long non-coding RNA (lncRNA), terminal differentiation-induced noncoding RNA (TINCR), colorectal carcinoma (CRC), microRNA-31 (miR-31)

## Abstract

Abnormal expression in terminal differentiation-induced noncoding RNA (TINCR), a long non-coding RNA (lncRNA), has been reported in different human cancers, including colorectal carcinoma (CRC). Moreover, the molecular mechanisms that underlie the effects of TINCR on CRC remain unclear. Here, by a set of bioinformatics studies, we found that microRNA-31 (miR-31), the oncogenic miRNA that robustly upregulates in CRC, was a sponge miRNA for TINCR. TINCR and miR-31 levels were inversely correlated in both CRC tissues and CRC cell lines. Luciferase reporter assay revealed a specific binding site on TINCR for miR-31. Suppression of TINCR promoted CRC cell growth and migration in vitro, while overexpression of TINCR inhibited CRC cell growth and migration in vitro. TINCR depletion increased tumor xenograft growth in vivo, while TINCR overexpression inhibited it. Together, our study suggests that re-expressing TINCR may suppress invasive outgrowth of CRC through miR-31.

## INTRODUCTION

Colorectal carcinoma (CRC) accounts for about 90% of all colorectal carcinoma cases [1], and is one of the leading reasons for cancer-related mortality [2]. Surgical removal of the primary tumor, endoscopic non-invasive treatment, radiotherapy, chemotherapy and radiation have now been co-applied during therapy, which has significantly improved the overall patient survival [3]. However, clinical outcomes of patients with CRC are still unsatisfactory and need further improvement [4, 5]. Therefore, better description and comprehension of the regulators of the aggressive manner of CRC are urgently required for developing novel therapeutic strategies in clinic.

Long non-coding RNAs (lncRNAs) are a group of endogenous and non-protein coding RNAs that are more than 200 nucleotides in length, and were first identified from sequencing and microarray analyses of the whole genome and transcriptome [6]. LncRNAs are functionally heterogeneous and abundant RNAs acting in all cellular compartments that can form complexes with DNA, RNA, and proteins. Accumulating evidence shows that lncRNAs are dysregulated in nearly all types of human cancer, and significantly influence diverse pathophysiological processes, including innate immunity, metabolism and carcinogenesis [7–9]. An increasing number of studies have indicated that long non-coding RNAs (lncRNAs) play important roles in tumorigenesis and tumor development [10–12]. Numerous lncRNAs are dysregulated in CRC, and are implicated in the genesis and development of CRC [13]. Accordingly, therapies that target lncRNAs might be attractive strategies for treating patients with CRC.

TINCR was initially discovered as a lncRNA required for induction of key differentiation genes in epidermal tissue, including genes mutated in human skin diseases characterized by disrupted epidermal barrier formation [14, 15]. Aberration in TINCR expression has been identified in multiple human cancer types, and its aberrant expression has been shown to have significant functions in cancer progression. TINCR has been reported involved in the tumorigenesis of many different human cancers [16–20], including CRC [21, 22]. However, the mechanisms that underlie the effects of TINCR on CRC remain unclear.

Here, by a set of bioinformatics studies, we found that microRNA-31 (miR-31), the oncogenic miRNA that robustly upregulates in CRC, was a sponge miRNA for TINCR. TINCR and miR-31 levels were inversely correlated in both CRC tissues and CRC cell lines. Luciferase reporter assay revealed a specific binding site on TINCR for miR-31. Suppression of TINCR promoted CRC cell growth and migration in vitro, while overexpression of TINCR inhibited CRC cell growth and migration in vitro. TINCR depletion increased tumor xenograft growth in vivo, while TINCR overexpression inhibited it. Together, our study suggests that re-expressing TINCR may suppress invasive outgrowth of CRC through miR-31.

## RESULTS

### miR-31 is the only TINCR-targeting miRNA that dramatically increases in CRC tissues

**Figure 1:**
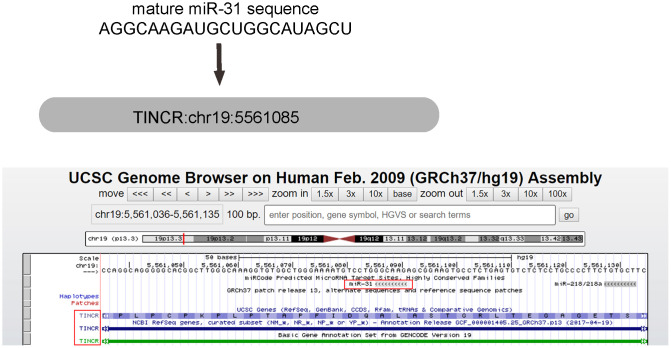
**miR-31 is the only TINCR-targeting miRNA that dramatically increases in CRC tissues.** MiR-31 is one special TINCR-targeting miRNA, analyzed by miRcode, with the binding region on TINCR was shown.

Using miRcode (http://www.mircode.org/mircode), we found that miR-31 is one special TINCR-targeting miRNA (Figure 1). Moreover, miR-31 is well known to dramatically upregulate in CRC and acts as an oncogenic miRNA through many genes [23–28]. Since TINCR was decreased in CRC [21], we thought that miR-31 may be a good candidate to study as a possible sponge miRNA for TINCR (Table 1).

**Table 1:** Reports on TINCR expression in different cancers.

**LncRNA name**	**Cancer name**	**Methods**	**Expression pattern**	**Pubmed ID**
TINCR	breast cancer	qPCR, Luciferase reporter assay, in vitro knockdown etc.	up-regulated	29614984
TINCR	lung cancer	qPCR, Western blot, Luciferase reporter assay, in vitro knockdown, RIP etc.	down-regulated	29324317
TINCR	non small cell lung cancer	Microarray, qPCR, RNAi, RIP etc.	up-regulated	29427662
TINCR	bladder cancer	qPCR, RNAi, Cell proliferation assay etc.	up-regulated	27586866
TINCR	colorectal cancer	qPCR, Western blot, RIP, Luciferase reporter assay, Luciferase reporter assay, Flow cytometry assay, Cell proliferation assay etc.	down-regulated	27009809
TINCR	esophageal squamous cell cancer	qPCR, RNAi, Flow cytometry assay, Cell proliferation assay etc.	up-regulated	26833746
TINCR	gastric cancer	qPCR	up-regulated	28569791
TINCR	gastric cancer	qPCR etc.	differential expression	27893425
TINCR	gastric cancer	qPCR, Western blot, Luciferase report assay etc.	up-regulated	28744139
TINCR	gastric cancer	microarray, qPCR etc.	up-regulated	28042329
TINCR	gastric cancer	RNA-seq, qPCR, RNAi, Western blot etc.	up-regulated	25728677

### TINCR and miR-31 levels inversely correlate in CRC tissues and cell lines

**Figure 2:**
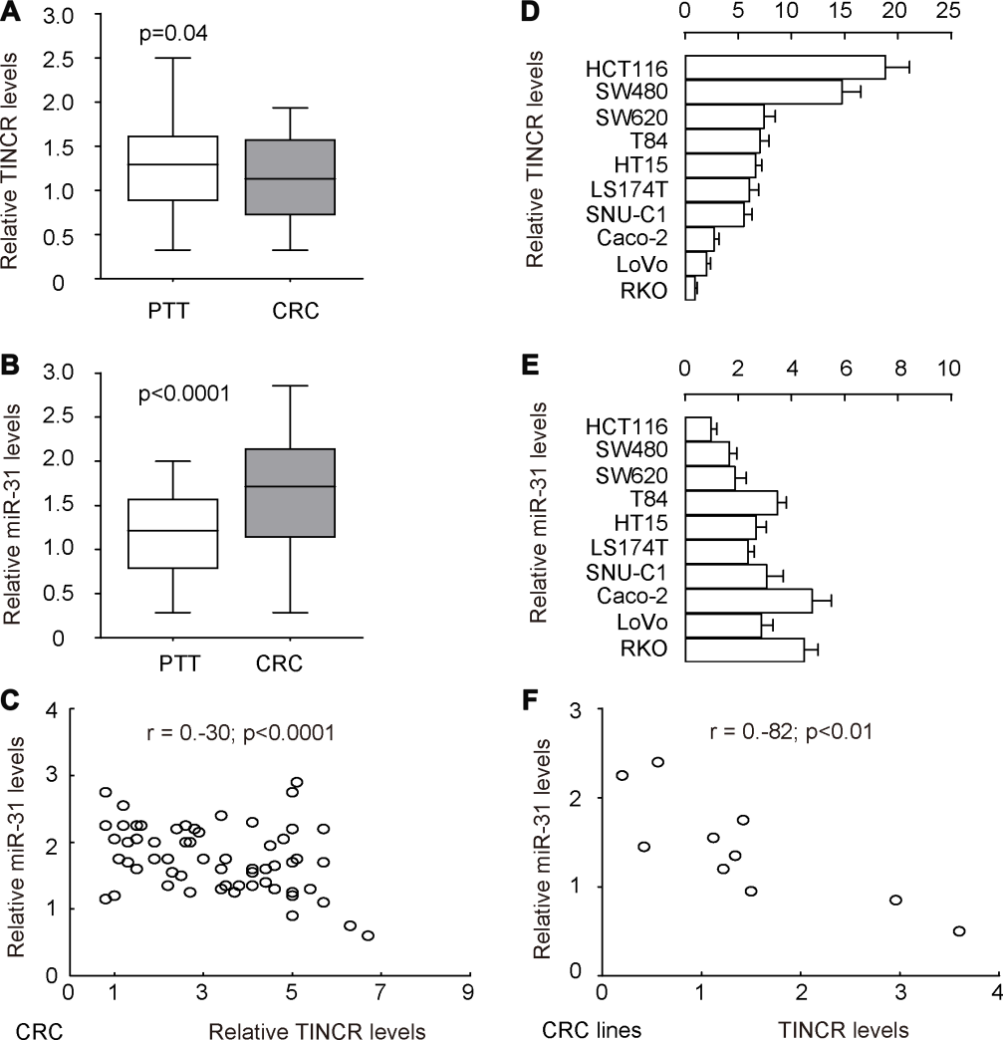
**TINCR and miR-31 levels inversely correlate in CRC tissues and cell lines.** To explore the potential functions of TINCR in the development of CRC, its expression pattern was investigated in 77 pairs of CRC tissues and peritumoral tissues (PTT). (**A**, **B**) RT-qPCR for TINCR (**A**) and miR-31 (**B**) in CRC tissues, compared to PTT. (**C**) Correlation test for TINCR and miR-31 in all 77 cases, which showed a significant inverse correlation (r=-0.3; p<0.0001). (**D**, **E**) RT-qPCR for TINCR (**D**) and miR-31 (**E**) in human CRC cell lines, including HCT116, SW480, SW620, T84, HT15, LS174T, SNU-C1, Caco-2, LoVo and RKO. (**F**) Correlation test for TINCR and miR-31 in all examined CRC cell lines, which showed a significant correlation (r=-0.82, p<0.01). Five repeats were done for cell lines.

To explore the potential functions of TINCR in the development of CRC, its expression pattern as well as association with metastasis was investigated in 77 pairs of CRC tissues and peritumoral tissues (PTT) (Table 2). Interestingly, the data of RT-qPCR revealed that TINCR was slightly but significantly reduced in CRC tissues, compared to PTT (Figure 2A, p=0.04). However, the increase in miR-31 levels in CRC tissues was dramatical and significant, compared to PTT (Figure 2B, p<0.0001). In addition, all 77 cases were put together, which showed a significant inverse correlation (r=-0.3; p<0.0001, Figure 2C). Further measurements of TINCR (Figure 2D) and miR-31 (Figure 2E) expression were done in different human CRC cell lines, including HCT116, SW480, SW620, T84, HT15, LS174T, SNU-C1, Caco-2, LoVo and RKO, for selecting proper cell lines for mechanistic studies. Of note, the levels of TINCR and miR-31 in these 10 cell lines also exhibited an inverse correlation (r=-0.82, p<0.01, Figure 2F). Together, these data suggest a possible functional interaction between TINCR and miR-31.

**Table 2:** Multivariate analysis for association of TINCR with clinicopathological parameters in CRC patients.

**Parameter**	**TINCR expression**		**P**
	**High**	**Low**	
**Age (years)**			0.25
< 60	19	18	
≥ 60	18	22	
**Tumor size (cm)**			0.035*
< 2.5	23	15	
≥ 2.5	14	25	
**Differentiated degree**			0.046*
G1+G2	22	18	
G3	15	22	
**FIGO stage**			0.064
I-II	20	17	
III-IV	17	23	
**Lymphatic metastasis**			0.018^*^
No	21	11	
Yes	16	29	
**Liver/lung metastasis**			0.04*
No	36	6	
Yes	1	34	

G1: Well differentiated; G2: Moderately differentiated; G3: Poorly differentiated.

### TINCR is a molecular sponge to interact with miR-31 in CRC

**Figure 3:**
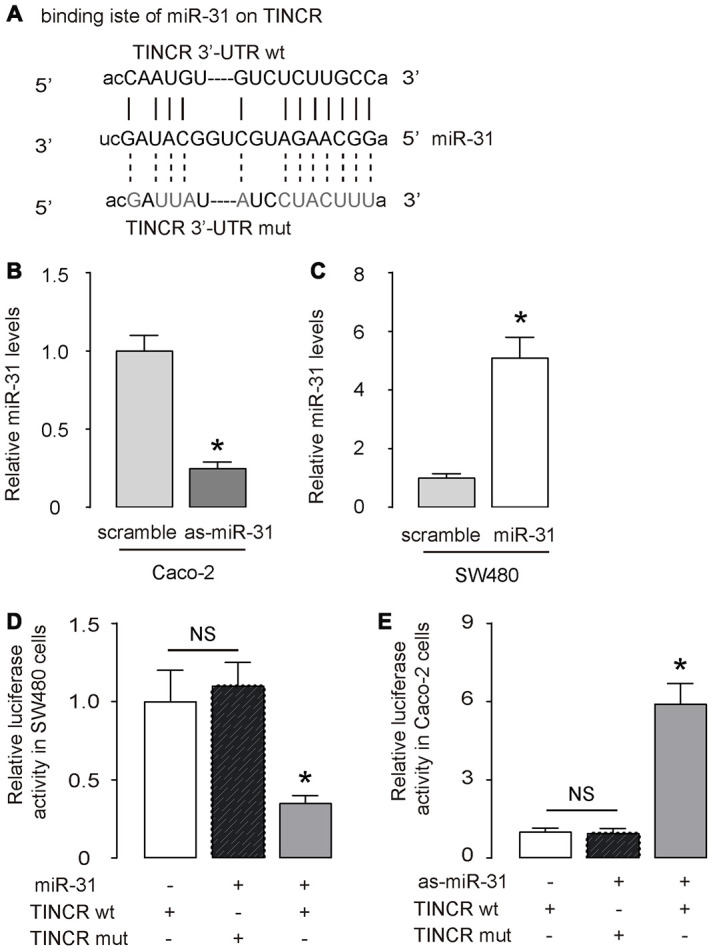
**TINCR is a molecular sponge to interact with miR-31 in CRC.** (**A**) The binding (site) between TINCR and miR-31 was predicted and shown by StarBase. (**B**, **C**) RT-qPCR for miR-31 in as-miR-31-transfected Caco-2 cells vs scrambled-transfected Caco-2 cells (B), and in miR-31-transfected SW480 cells vs scrambled-transfected SW480 cells (**C**). (**D**, **E**) A luciferase reporter assay was done to examine whether TINCR could interact with miR-31 in SW480 cells by co-transfection of miR-31 with TINCR-wt or TINCR-mut (**D**), and to examine whether TINCR could interact with miR-31 in Caco-2 cells by co-transfection of as-miR-31 with TINCR-wt or TINCR-mut (**E**). N=5. *p<0.05. NS: non-significant.

Next, the binding (site) between TINCR and miR-31 was predicted by StarBase (http://starbase.sysu.edu.cn/) (Figure 3A). We thus prepared luciferase reporter for wildtype (wt) TINCR and TINCR with a mutant at the miR-31 binding site (mut) (Figure 3A). CaCO2 cells expressed relatively high miR-31 and low TINCR (Figure 2D, 2E), and thus were selected as the cell line for depleting miR-31 or for overexpressing TINCR. SW480 cells expressed relatively low miR-31 and high TINCR (Figure 2D, 2E), and thus were selected as the cell line for overexpressing miR-31 or for depleting TINCR. First, transfection with as-miR-31 significantly reduced miR-31 levels in Caco-2 cells (Figure 3B), while transfection with miR-31 significantly increased miR-31 levels in SW480 cells (Figure 3C). LncRNA can serve as a molecular sponge to interact with miRNAs [29]. Thus, we performed a luciferase reporter assay to examine whether TINCR could interact with miR-31 in CRC cells. In SW480 cells, the transfection of miR-31 markedly reduced the luciferase activity of TINCR-wt (p<0.05), whereas the luciferase activity of the TINCR-mut was unaffected upon miR-31 upregulation (Figure 3D). Moreover, in Caco-2 cells, the transfection of as-miR-31 markedly increased the luciferase activity of TINCR-wt (p<0.05), whereas the luciferase activity of the TINCR-mut was unaffected upon miR-31 downregulation (Figure 3E). Together, these data suggest that TINCR is a molecular sponge to interact with miR-31 in CRC.

### Overexpression or depletion of TINCR in CRCs

**Figure 4:**
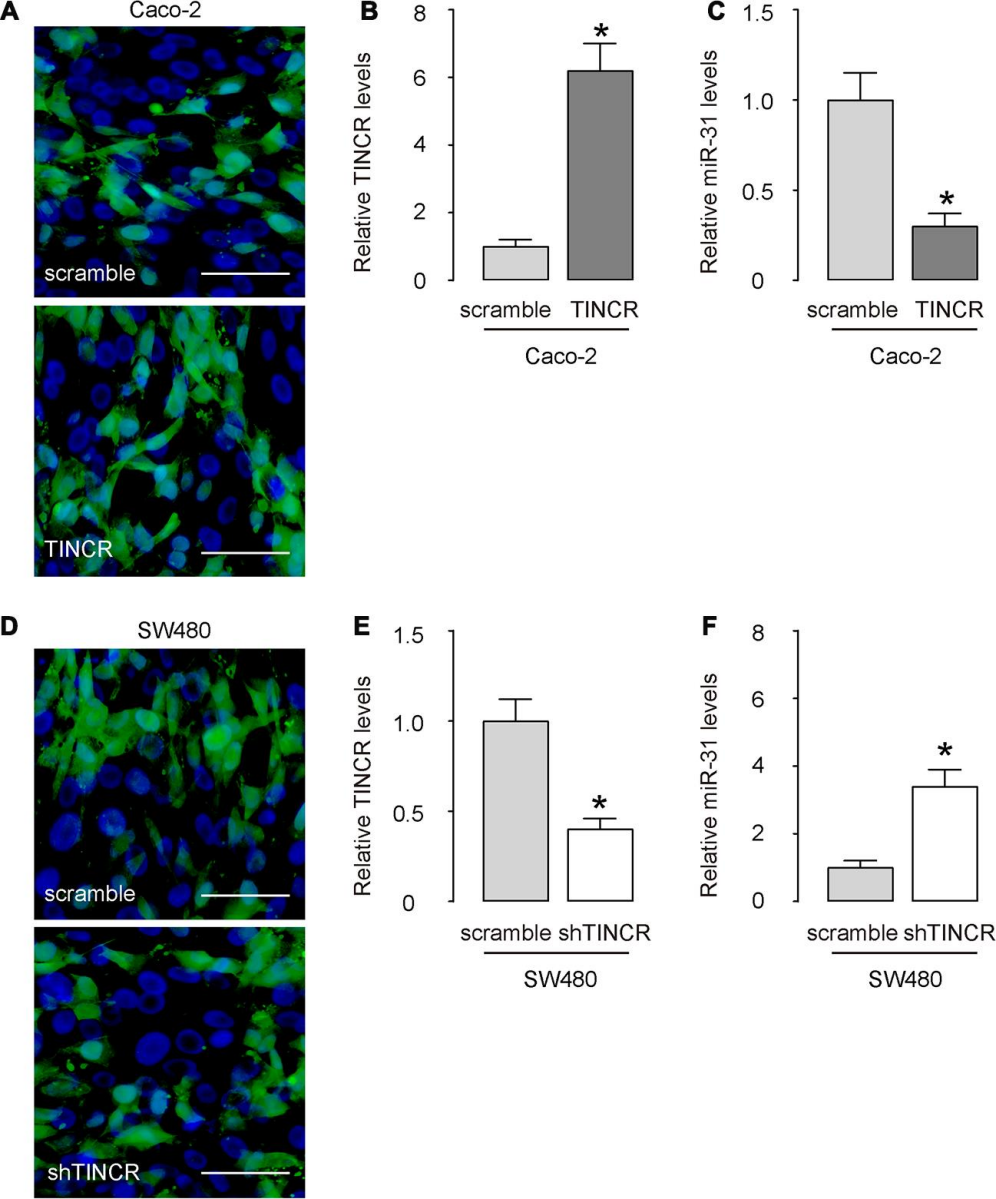
**Overexpression or depletion of TINCR in CRCs.** (**A**–**C**) Transfection with TINCR or scramble in Caco-2 cells, shown by representative cell images in culture (**A**), and by RT-qPCR for TINCR (**B**) and for miR-31 (**C**). (**D**, **E**) Transfection with shTINCR or scramble in SW480 cells, shown by representative cell images in culture (**D**), and by RT-qPCR for TINCR (**E**) and for miR-31 (**F**). N=5. *p<0.05. Scale bars are 20μm.

Next, we prepared plasmids that overexpress or deplete TINCR in CRCs. Transfection with TINCR significantly increased TINCR levels in Caco-2 cells (Figure 4A, 4B), while transfection with shTINCR significantly decreased TINCR levels in SW480 cells (Figure 4C, 4D).

### Suppression of TINCR promotes CRC cell growth in vitro without affecting cell apoptosis

**Figure 5:**
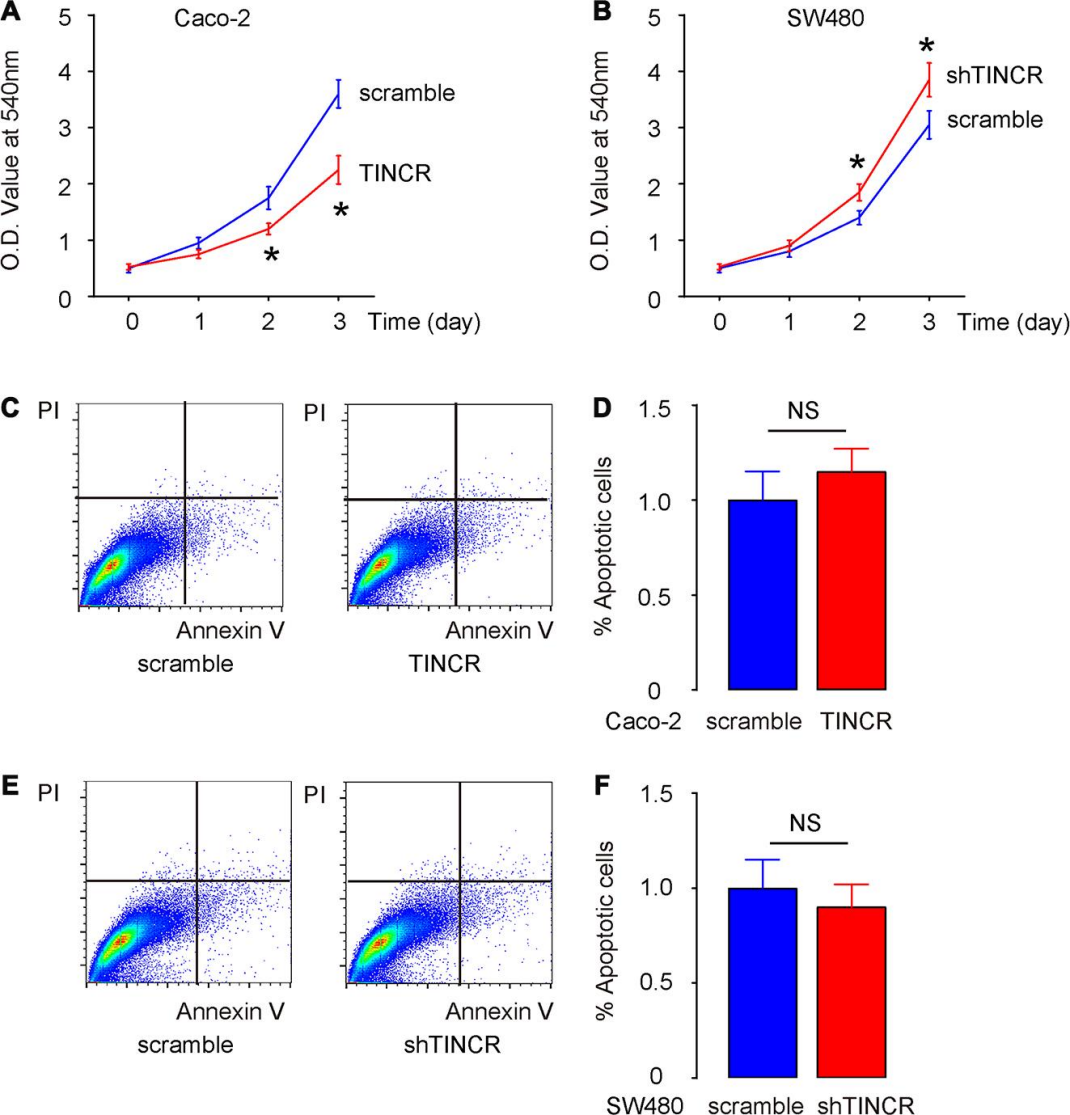
**Suppression of TINCR promotes CRC cell growth in vitro without affecting cell apoptosis.** (**A**, **B**) The effects of TINCR modification on Caco-2 (**A**) and SW480 (**B**) cell growth were examined in vitro by CCK-8 assay. (**C**, **D**) Cell apoptosis was examined in Caco-2 cells by Annexin V/PI-based flow cytometry assay, shown by representative flow charts (**C**) and by quantification (**D**). (**E**, **F**) Cell apoptosis was examined in SW480 cells by Annexin V/PI-based flow cytometry assay, shown by representative flow charts (**E**) and by quantification (**F**). N=5. *p<0.05. NS: non-significant.

Then, we examined the effects of TINCR modification on CRC cell growth in vitro. First, the CCK-8 assay was performed to evaluate the influence of TINCR overexpression or depletion on CRC cell growth. The absorbance was significantly lower in TINCR-transfected Caco-2 cells (Figure 5A), and significantly higher in shTINCR-transfected SW480 cells (Figure 5B). However, neither overexpression of TINCR in Caco-2 cells (Figure 5C, 5D), nor depletion of TINCR in SW480 cells (Figure 5E, 5F), appeared to significantly alter apoptosis. Thus, the effects of TINCR on CRC growth should be mediated through cell proliferation, rather than through cell apoptosis.

### Suppression of TINCR promotes CRC cell metastasis in vitro

**Figure 6:**
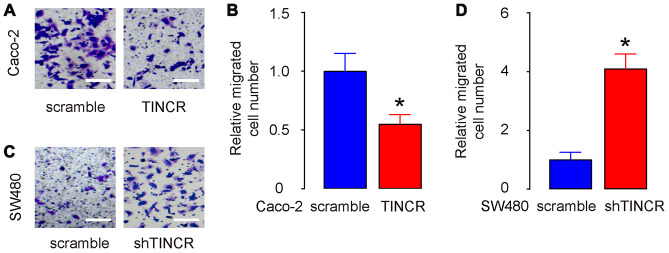
**Suppression of TINCR promotes CRC cell metastasis in vitro.** (**A**, **B**) The migration of TINCR-transfected Caco-2 cells was then examined in a transwell migration assay, shown by representative images (**A**) and by quantification (**B**). (**C**, **D**) The migration of shTINCR-transfected SW480 cells was then examined in a transwell migration assay, shown by representative images (**C**) and by quantification (**D**). N=5. *p<0.05.

The migration of CRC cells in vitro was then examined in TINCR-modified CRC cells. The transwell migration assay showed that the overexpression of TINCR suppressed the migratory ability of Caco-2 cells, shown by representative images (Figure 6A) and by quantification (p<0.05, Figure 6B). On the other hand, depletion of TINCR increased the migratory ability of SW480 cells, shown by representative images (Figure 6C) and by quantification (p<0.05, Figure 6D). Thus, suppression of TINCR promotes CRC cell metastasis in vitro.

### Overexpression of TINCR inhibits CRC growth in vivo

**Figure 7:**
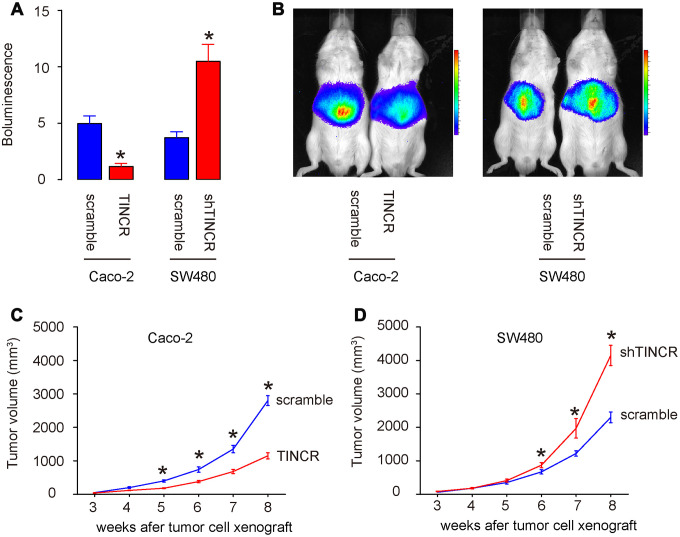
**Overexpression of TINCR inhibits CRC growth in vivo.** In vivo xenograft experiments (subcutaneous transplantation with lentivectors carrying luciferase reporter and shTINCR/TINCR/scramble) were conducted to analyze the role of TINCR on tumor growth *in vivo*. (**A**, **B**) At 8 weeks after xenografting, tumor growth was determined by bioluminescence, shown by quantification (**A**), and by representative images (**B**). (**C**, **D**) Tumor growth curve after xenografting of Caco-2 cells (**C**) and SW480 cells (**D**). N=5. *p<0.05.

*In vivo* xenograft experiments were conducted to analyze the role of TINCR on tumor growth *in vivo*. Caco-2 cells transduced with lentivirus carrying TINCR or scramble with luciferase, and SW480 cells transduced with lentivirus carrying shTINCR or scramble with luciferase, were subcutaneously injected into the NOD/SCID mice. The formation of xenograft-derived tumor was measured every week. After 8 weeks, the quantification of tumor formation in living animals was performed using bioluminescence detection system. The increase in TINCR expression inhibited Caco-2 tumor cell growth in vivo, shown by quantification (p<0.05, Figure 7A), and by representative images (Figure 7B). While the decrease in TINCR expression enhanced SW480 tumor cell growth in vivo, shown by quantification (p<0.05, Figure 7A), and by representative images (Figure 7B). These data were consistent with the weekly measurement of tumor volume (Figure 7C–7D). Collectively, the overexpression of TINCR inhibits CRC growth through suppression of miR-31.

## DISCUSSION

An increasing number of studies have demonstrated the important regulatory roles of lncRNAs in carcinogenesis and cancer progression [30–32]. A variety of lncRNAs are aberrantly expressed in CRC, and play dispensable roles in regulating a wide range of biological activities, such as cell proliferation, cycle, apoptosis, metastasis, and epithelial-mesenchymal transition [6]. Therefore, identification of the detailed roles of lncRNAs in CRC pathogenesis might facilitate the development of effective targets for the treatment of CRC patients.

TINCR expression is also reduced in prostate [16] and lung [17] cancers. For example, prostate cancer patients with low TINCR expression exhibit a poorer prognosis than those with high TINCR expression [16]. In contrast, TINCR is upregulated in hepatocellular carcinoma, and high TINCR expression is significantly correlated with tumor size, tumor differentiation, TNM stage, and vascular invasion [18]. Hepatocellular carcinoma patients with high TINCR expression have shorter disease-free survival and overall survival than those with low TINCR expression [18]. Increased expression of TINCR is also observed in breast [19] and gastric [20] cancers.

Previous studies have shown that insufficient TINCR expression promotes proliferation, metastasis through activating EpCAM cleavage in CRCs [21, 22]. Here, we found that TINCR is a sponge for miR-31. Given that miR-31 was shown to target ARID1A to further transactivate EpCAM and subseqently enhance the oncogenicity and stemness of head and neck squamous cell carcinoma [33], it is highly possible that miR-31 is the mediator for the TINCR-regulated EpCAM, reported by Zhang et al. [21]. In a recent study, TINCR was however found to increase in CRC and seemed to work as a sponge for tumor suppressor miR-7 [34]. To figure out the possible reason for this discrepancy, we tried to surf TCGA database. However, our approach failed due to poor availability of the raw data from these studies, which prevented a deep analysis on the relationship between TINCR and interested miRNAs. On the other hand, the patients’ information provided in the two studies did not show significant difference in the selection of tumor stage and malignancy. Thus, we think that the only reason for this discrepancy of TINCR levels may be due to regional difference in the participated cases, as people from these two regions had major difference in appetite, which could affect the delicate molecular characteristics of the CRCs.

Here, we provided a set of strong evidence to demonstrate the interaction between TINCR and miR-31. First, we detected an obvious inverse correlation between TINCR and miR-31 in not only the clinical CRC specimens, but also in selected commonly used CRC lines. Second, bioinformatic studies demonstrated the binding site for miR-31 on TINCR, which was proved to be functional in a luciferase reporter assay. Third, overexpressing TINCR in CRCs decreased miR-31, while depleting TINCR increased miR-31.

Next, we found that suppression of TINCR promoted CRC cell growth and migration in vitro, without affecting cell apoptosis. Overexpression of TINCR inhibited CRC cell growth and migration in vitro, without affecting cell apoptosis. TINCR depletion increased tumor growth in vivo, while TINCR overexpression inhibited tumor growth in vivo. The function of TINCR in CRC appeared to be tumor-suppressive, which is different from how it functioned in gastric cancer, breast cancer and hepatocellular carcinoma. These likely inconsistent results in different cancers may result from the difference in expression of the interactive partners of TINCR in different cancers, e.g. certain miRNAs functioning as sponge miRNAs or certain genes and their mRNAs [7–9].

The effects of TINCR on CRC growth appeared not to be attributable to cell apoptosis. Since cell growth is a summary of cell proliferation and cell death, it is expected that increase in CRC cell growth by TINCR depletion could be primarily resulting from increases in cell proliferation. Of note, the depletion of TINCR on increases in cell metastasis could help to detach cell junctions and increase the intercellular spaces to promote cell proliferation [35]. Thus, the effect of TINCR on metastasis may be more important than its direct effect on cell proliferation in CRC.

The miR-31 is a well-known oncogenic miRNA in many cancers [23–28], including CRC [36]. It has multiple determined target genes and the net effect is tumor promoting [23–28, 36]. Hence, in the current study, we did not look at its downstream genes, since it is not the key question here. In summary, our study showed that TINCR is an endogenous sponge of miR-31, while the decrease in TINCR expression increases the miR-31 expression, thereby enhancing tumor growth in CRC. Our research provides novel data regarding the mechanisms underlying CRC pathogenesis, and may be helpful in identifying promising novel targets for the treatment of CRC.

## MATERIALS AND METHODS

### Protocol approval, patients and mice

The current study and animal work were approved by the Ethics Committee of Zhongshan Hospital of Fudan University and carried out in accordance with the Declaration of Helsinki. Written informed consent was provided by all the enrolled patients before their participation into the research. In total, 77 pairs of CRC tissues and adjacent normal tissues were collected from patients at Zhongshan Hospital of Fudan University. Immediately after surgical resection, all these tissue specimens were snap frozen in liquid nitrogen and then stored at −80°C until further use. CRC patients who had been treated with chemotherapy or radiotherapy prior to surgical resection were excluded from the research.

### Cell culture

The human CRC cell lines, including HCT116, SW480, SW620, T84, HT15, LS174T, SNU-C1, Caco-2, LoVo and RKO, were all purchased from American Type Culture Collection (ATCC, Rockville, MD, USA). The cells from all these cell lines were cultured in Dulbecco's modified Eagle's medium (DMEM; Gibco; Thermo Fisher Scientific, Inc., Waltham, MA, USA) containing 10% fetal bovine serum (FBS; Gibco; Thermo Fisher Scientific, Inc.), 100 U/mL penicillin (Sigma-Aldrich; Merck KGaA, Darmstadt, Germany), and 100 mg/mL streptomycin (Sigma-Aldrich, St Louis, MO, USA). The cell cultures were maintained at 37°C in a humidified atmosphere under 5% CO_2_ conditions.

### Bioinformatics analysis and luciferase reporter assay

The binding (site) between TINCR and miR-31 was predicted by miRcode (http://www.mircode.org/mircode) and StarBase (http://starbase.sysu.edu.cn/). The fragments of TINCR containing the predicted wild-type (wt) and mutant (mut) miR-31-binding sites were cloned into pmirGLO reporter vectors (Promega Corporation, Madison, WI, USA) to generate the TINCR-wt and TINCR-mut plasmids, respectively. For the reporter assay, cells were seeded into 24-well plates one day before transfection. The generated luciferase reporter plasmids, along with the miR-31 or antisense for miR-31 or scramble, were transfected into cells using Lipofectamine 2000. The transfected cells were collected after 48 h of transfection and subjected to the dual luciferase reporter assay (Promega) for the measurement of the luciferase activity. Firefly luciferase activity was normalized to the level of Renilla luciferase activity.

### Transfection and transduction

Small hairpin interfering RNAs (shRNA) against TINCR (shTINCR), TINCR and nontargeting control shRNA (scramble) used a pcDNA3.1-CMV-GFP plasmid as backbone (Clontech, Mountain View, CA, USA). Cells were plated into six-well plates at a density of 5 × 10^5^ cells per well. Cell transfection was conducted with Lipofectamine 2000 (Invitrogen, Carlsbad, USA) according to the protocol recommended by the manufacturer. For in vivo xenograft experiments, plasmids that co-express luciferase and TINCR/shTINCR/scramble were used to prepare lentiviral vectors. Seeded HEK293T cells (ATCC) were co-transfected with 5 μg of prepared plasmids and 5 μg each of packaging plasmids (REV, pMDL and VSV-G) by Lipofectamine-2000. The supernatant was removed 48 hours after transfection and filtered through the 0.45 μm syringe filter, after which the lentivirus in supernatant was further processed, isolated and titrated. For *in vitro* transduction, a multiplicity of infection (MOI) of 100 was used and the incubation time was 48 hours to allow completeness of viral infection.

### Extraction of total RNA and reverse transcription-quantitative polymerase chain reaction (RT-qPCR)

The extraction of total RNA was performed with a high-purity total RNA extraction kit (BioTeke Ltd., Beijing, China). Total RNA was reverse transcribed using a miScript Reverse Transcription kit (Qiagen GmbH, Hilden, Germany), according to the manufacturer’s protocol. The cDNA was then used for the detection of miR-31 expression using a miScript SYBR Green PCR kit (Qiagen GmbH). the synthesis of cDNA was conducted using a PrimeScript first-strand cDNA synthesis kit (Takara Biotechnology Co., Ltd., Dalian, China); the cDNA was then subjected to qPCR using a SYBR Premix ExTaq kit (Takara Biotechnology Co.). The expression of miR-31 was normalized to glyceraldehyde phosphate dehydrogenase (GAPDH). Relative gene expression was calculated using the 2^–ΔΔCq^ method.

### Cell counting kit-8 (CCK-8) assay

The transfected cells were collected after 24 h of incubation and suspended in complete culture medium. A total of 100μl of each suspension containing 2000 cells was seeded into 96-well plates. Cell proliferation was evaluated using the CCK-8 assay (Dojindo, Kumamoto, Japan) at four time points (0, 1, 2, and 3 days after incubation). For this assay, 10 μl of CCK-8 solution was added to the cells, followed by incubation at 37°C for an additional 2 h. The absorbance of the samples at a wavelength of 450 nm was measured using the VarioskanTM LUX microplate reader (Thermo Fisher Scientific).

### Analysis of apoptosis by flow cytometry

The apoptosis rate was determined using an Annexin V fluorescein isothiocyanate (FITC) apoptosis detection kit (Biolegend, San Diego, CA, USA), referring to the protocols recommended by the manufacturer. After 48 h of culture, the transfected cells were collected and washed thrice with ice-cold phosphate buffer solution (PBS; Gibco; Thermo Fisher Scientific, Inc.). The transfected cells were then double-stained with 5 μl of Annexin V and 5 μL of PI diluted in 100 μl of binding buffer. Following incubation for 30 min in the dark, flow cytometry (FACScan; BD Biosciences, Bedford, MA, USA) was performed for the determination of the apoptotic condition of cells.

### Transwell migration assay

At 48 h post-transfection, the cells were washed thrice with PBS and suspended in FBS-free DMEM. In total, 200 μL of cell suspension containing 5 × 10^4^ transfected cells was plated into the upper compartments of transwell inserts (8 μM pore size, Costar, Cambridge, MA, USA). The bottom compartments were covered with 500 μL of DMEM containing 20% FBS; this medium acts as a chemoattractant. Following incubation for 24 h, the non-migrating cells in the upper compartment were gently removed with a cotton swab, whereas the migrating cells were fixed in 4% paraformaldehyde and stained with 0.5% crystal violet. The migration of the cells was assessed by counting the average number of migrated cells in six randomly selected fields of each insert under an IX83 inverted microscope (Olympus Corporation, Tokyo, Japan).

### In vivo xenograft experiments

Caco-2 cells transduced with lentivirus carrying TINCR or scramble with luciferase, and SW480 cells transduced with lentivirus carrying shTINCR or scramble with luciferase, were subcutaneously injected into the NOD/SCID mice (Shanghai SLAC Laboratory Animal Co. Ltd., Shanghai, China). The formation of xenograft-derived tumor was measured every week. After 8 weeks, the quantification of tumor formation in living animals was performed using bioluminescence detection system (IVIS imaging system, Xenogen Corp., Alameda, CA, USA), 10 minutes after intraperitoneal injection of luciferin at 150 mg/kg body weight. The acquisition time was set to 1 minute and the binning value was 10.

### Statistical analysis

The statistical analysis was performed with the GraphPad Prism 7 (GraphPad Software, San Diego, CA, USA). All results were expressed as the mean ± standard deviation (SD) from at least 5 independent experiments. All values represent the mean ± standard deviation (SD). The multivariate analysis was performed between TINCR and different clinicopathological characteristics in patients with CRC. The comparison between two groups was examined using the two-tailed Student’s t-test, while one-way analysis of variance (ANOVA) followed by a Dunnett’s post hoc test was used to determine the differences among multiple groups. The correlation between the expression of TINCR and miR-31 was evaluated by Spearman’s correlation analysis. P values < 0.05 were deemed statistically significant.

## References

[1] Lupia M, Cavallaro U. Ovarian cancer stem cells: still an elusive entity? Mol Cancer. 2017; 16:64. 10.1186/s12943-017-0638-3 28320418 PMC5360065

[2] Garza-Treviño EN, Said-Fernández SL, Martínez-Rodríguez HG. Understanding the colon cancer stem cells and perspectives on treatment. Cancer Cell Int. 2015; 15:2. 10.1186/s12935-015-0163-7 25685060 PMC4328053

[3] De Maio G, Zama E, Rengucci C, Calistri D. What influences preneoplastic colorectal lesion recurrence? Oncotarget. 2017; 8:12406–16. 10.18632/oncotarget.13628 27902488 PMC5355354

[4] La Vecchia C. Ovarian cancer: epidemiology and risk factors. Eur J Cancer Prev. 2017; 26:55–62. 10.1097/CEJ.0000000000000217 26731563

[5] Candido-dos-Reis FJ, Song H, Goode EL, Cunningham JM, Fridley BL, Larson MC, Alsop K, Dicks E, Harrington P, Ramus SJ, de Fazio A, Mitchell G, Fereday S, et al, and EMBRACE, and kConFab Investigators, and Australian Ovarian Cancer Study Group. Germline mutation in BRCA1 or BRCA2 and ten-year survival for women diagnosed with epithelial ovarian cancer. Clin Cancer Res. 2015; 21:652–57. 10.1158/1078-0432.CCR-14-2497 25398451 PMC4338615

[6] Birney E, Stamatoyannopoulos JA, Dutta A, Guigó R, Gingeras TR, Margulies EH, Weng Z, Snyder M, Dermitzakis ET, Thurman RE, Kuehn MS, Taylor CM, Neph S, et al, and ENCODE Project Consortium, and NISC Comparative Sequencing Program, and Baylor College of Medicine Human Genome Sequencing Center, and Washington University Genome Sequencing Center, and Broad Institute, and Children’s Hospital Oakland Research Institute. Identification and analysis of functional elements in 1% of the human genome by the ENCODE pilot project. Nature. 2007; 447:799–816. 10.1038/nature05874 17571346 PMC2212820

[7] Gutschner T, Diederichs S. The hallmarks of cancer: a long non-coding RNA point of view. RNA Biol. 2012; 9:703–19. 10.4161/rna.20481 22664915 PMC3495743

[8] Zhang X, Gejman R, Mahta A, Zhong Y, Rice KA, Zhou Y, Cheunsuchon P, Louis DN, Klibanski A. Maternally expressed gene 3, an imprinted noncoding RNA gene, is associated with meningioma pathogenesis and progression. Cancer Res. 2010; 70:2350–58. 10.1158/0008-5472.CAN-09-3885 20179190 PMC2987571

[9] Yu G, Yao W, Gumireddy K, Li A, Wang J, Xiao W, Chen K, Xiao H, Li H, Tang K, Ye Z, Huang Q, Xu H. Pseudogene PTENP1 functions as a competing endogenous RNA to suppress clear-cell renal cell carcinoma progression. Mol Cancer Ther. 2014; 13:3086–97. 10.1158/1535-7163.MCT-14-0245 25249556 PMC4265235

[10] Luo M, Li Z, Wang W, Zeng Y, Liu Z, Qiu J. Long non-coding RNA H19 increases bladder cancer metastasis by associating with EZH2 and inhibiting e-cadherin expression. Cancer Lett. 2013; 333:213–21. 10.1016/j.canlet.2013.01.033 23354591

[11] Lai MC, Yang Z, Zhou L, Zhu QQ, Xie HY, Zhang F, Wu LM, Chen LM, Zheng SS. Long non-coding RNA MALAT-1 overexpression predicts tumor recurrence of hepatocellular carcinoma after liver transplantation. Med Oncol. 2012; 29:1810–16. 10.1007/s12032-011-0004-z 21678027

[12] Matouk IJ, Mezan S, Mizrahi A, Ohana P, Abu-Lail R, Fellig Y, Degroot N, Galun E, Hochberg A. The oncofetal H19 RNA connection: hypoxia, p53 and cancer. Biochim Biophys Acta. 2010; 1803:443–51. 10.1016/j.bbamcr.2010.01.010 20117150

[13] Kalmár A, Nagy ZB, Galamb O, Csabai I, Bodor A, Wichmann B, Valcz G, Barták BK, Tulassay Z, Igaz P, Molnár B. Genome-wide expression profiling in colorectal cancer focusing on lncRNAs in the adenoma-carcinoma transition. BMC Cancer. 2019; 19:1059. 10.1186/s12885-019-6180-5 31694571 PMC6836529

[14] Kretz M. TINCR, staufen1, and cellular differentiation. RNA Biol. 2013; 10:1597–601. 10.4161/rna.26249 24019000 PMC3866239

[15] Kretz M, Siprashvili Z, Chu C, Webster DE, Zehnder A, Qu K, Lee CS, Flockhart RJ, Groff AF, Chow J, Johnston D, Kim GE, Spitale RC, et al. Control of somatic tissue differentiation by the long non-coding RNA TINCR. Nature. 2013; 493:231–35. 10.1038/nature11661 23201690 PMC3674581

[16] Dong L, Ding H, Li Y, Xue D, Liu Y. LncRNA TINCR is associated with clinical progression and serves as tumor suppressive role in prostate cancer. Cancer Manag Res. 2018; 10:2799–807. 10.2147/CMAR.S170526 30154672 PMC6108330

[17] Liu X, Ma J, Xu F, Li L. TINCR suppresses proliferation and invasion through regulating miR-544a/FBXW7 axis in lung cancer. Biomed Pharmacother. 2018; 99:9–17. 10.1016/j.biopha.2018.01.049 29324317

[18] Tian F, Xu J, Xue F, Guan E, Xu X. TINCR expression is associated with unfavorable prognosis in patients with hepatocellular carcinoma. Biosci Rep. 2017; 37:BSR20170301. 10.1042/BSR20170301 28546230 PMC5529205

[19] Liu Y, Du Y, Hu X, Zhao L, Xia W. Up-regulation of ceRNA TINCR by SP1 contributes to tumorigenesis in breast cancer. BMC Cancer. 2018; 18:367. 10.1186/s12885-018-4255-3 29614984 PMC5883880

[20] Chen Z, Liu H, Yang H, Gao Y, Zhang G, Hu J. The long noncoding RNA, TINCR, functions as a competing endogenous RNA to regulate PDK1 expression by sponging miR-375 in gastric cancer. Onco Targets Ther. 2017; 10:3353–62. 10.2147/OTT.S137726 28744139 PMC5513873

[21] Zhang ZY, Lu YX, Zhang ZY, Chang YY, Zheng L, Yuan L, Zhang F, Hu YH, Zhang WJ, Li XN. Loss of TINCR expression promotes proliferation, metastasis through activating EpCAM cleavage in colorectal cancer. Oncotarget. 2016; 7:22639–49. 10.18632/oncotarget.8141 27009809 PMC5008388

[22] Zhang X, Yao J, Shi H, Gao B, Zhang L. LncRNA TINCR/microRNA-107/CD36 regulates cell proliferation and apoptosis in colorectal cancer via PPAR signaling pathway based on bioinformatics analysis. Biol Chem. 2019; 400:663–75. 10.1515/hsz-2018-0236 30521471

[23] Cui Q. Significance of miR-27a and miR-31 in early diagnosis and prognosis of colorectal cancer. Oncol Lett. 2019; 18:3092–96. 10.3892/ol.2019.10621 31452786 PMC6676396

[24] Peng H, Wang L, Su Q, Yi K, Du J, Wang Z. MiR-31-5p promotes the cell growth, migration and invasion of colorectal cancer cells by targeting NUMB. Biomed Pharmacother. 2019; 109:208–16. 10.1016/j.biopha.2018.10.048 30396078

[25] Yang X, Xu X, Zhu J, Zhang S, Wu Y, Wu Y, Zhao K, Xing C, Cao J, Zhu H, Li M, Ye Z, Peng W. miR-31 affects colorectal cancer cells by inhibiting autophagy in cancer-associated fibroblasts. Oncotarget. 2016; 7:79617–28. 10.18632/oncotarget.12873 27793031 PMC5346740

[26] Schee K, Boye K, Abrahamsen TW, Fodstad Ø, Flatmark K. Clinical relevance of microRNA miR-21, miR-31, miR-92a, miR-101, miR-106a and miR-145 in colorectal cancer. BMC Cancer. 2012; 12:505. 10.1186/1471-2407-12-505 23121918 PMC3519622

[27] Xu XM, Qian JC, Deng ZL, Cai Z, Tang T, Wang P, Zhang KH, Cai JP. Expression of miR-21, miR-31, miR-96 and miR-135b is correlated with the clinical parameters of colorectal cancer. Oncol Lett. 2012; 4:339–45. 10.3892/ol.2012.714 22844381 PMC3402725

[28] Slaby O, Svoboda M, Fabian P, Smerdova T, Knoflickova D, Bednarikova M, Nenutil R, Vyzula R. Altered expression of miR-21, miR-31, miR-143 and miR-145 is related to clinicopathologic features of colorectal cancer. Oncology. 2007; 72:397–402. 10.1159/000113489 18196926

[29] Chan JJ, Tay Y. Noncoding RNA:RNA regulatory networks in cancer. Int J Mol Sci. 2018; 19:1310. 10.3390/ijms19051310 29702599 PMC5983611

[30] Yu WD, Wang H, He QF, Xu Y, Wang XC. Long noncoding RNAs in cancer-immunity cycle. J Cell Physiol. 2018; 233:6518–23. 10.1002/jcp.26568 29574911 PMC13482439

[31] Vallone C, Rigon G, Gulia C, Baffa A, Votino R, Morosetti G, Zaami S, Briganti V, Catania F, Gaffi M, Nucciotti R, Costantini FM, Piergentili R, et al. Non-coding RNAs and endometrial cancer. Genes (Basel). 2018; 9:187. 10.3390/genes9040187 29596364 PMC5924529

[32] Chen X, Sun Y, Cai R, Wang G, Shu X, Pang W. Long noncoding RNA: multiple players in gene expression. BMB Rep. 2018; 51:280–89. 10.5483/bmbrep.2018.51.6.025 29636120 PMC6033065

[33] Lu WC, Liu CJ, Tu HF, Chung YT, Yang CC, Kao SY, Chang KW, Lin SC. miR-31 targets ARID1A and enhances the oncogenicity and stemness of head and neck squamous cell carcinoma. Oncotarget. 2016; 7:57254–67. 10.18632/oncotarget.11138 27528032 PMC5302987

[34] Yu S, Wang D, Shao Y, Zhang T, Xie H, Jiang X, Deng Q, Jiao Y, Yang J, Cai C, Sun L. SP1-induced lncRNA TINCR overexpression contributes to colorectal cancer progression by sponging miR-7-5p. Aging (Albany NY). 2019; 11:1389–403. 10.18632/aging.101839 30853664 PMC6428101

[35] Díaz-Coránguez M, Liu X, Antonetti DA. Tight junctions in cell proliferation. Int J Mol Sci. 2019; 20:5972. 10.3390/ijms20235972 31783547 PMC6928848

[36] Nagy ZB, Wichmann B, Kalmár A, Galamb O, Barták BK, Spisák S, Tulassay Z, Molnár B. Colorectal adenoma and carcinoma specific miRNA profiles in biopsy and their expression in plasma specimens. Clin Epigenetics. 2017; 9:22. 10.1186/s13148-016-0305-3 28289479 PMC5310023

